# Chronic Headache Education and Self-Management Study (CHESS): a process evaluation

**DOI:** 10.1186/s12883-022-02792-1

**Published:** 2023-01-07

**Authors:** David R. Ellard, Vivien P. Nichols, Frances E. Griffiths, Martin Underwood, Stephanie J. C. Taylor, On behalf of the CHESS team (Consortium), David R. Ellard, Vivien P. Nichols, Frances E. Griffiths, Martin Underwood, Stephanie J. C. Taylor, Felix Achana, Dawn Carnes, Sandra Eldridge, Kirstie Haywood, Siew Wan Hee, Helen Higgins, Dipesh Mistry, Hema Mistry, Sian Newton, Chloe Norman, Ms Emma Padfield, Shilpa Patel, Stavros Petrou, Tamar Pincus, Rachel Potter, Harbinder Sandhu, Kimberley Stewart, Manjit Matharu

**Affiliations:** 1 Warwick Clinical Trials Unit, Warwick Medical School, University of Warwick, Coventry, CV4 7AL UK; 2 University Hospitals Coventry and Warwickshire, Clifford Bridge Road, Coventry, CV2 2DX UK; 3 Division of Health Sciences, Warwick Medical School, University of Warwick, Coventry, CV4 7AL UK; 4 Wolfson Institute of Population Health, Barts and The London School of Medicine and Dentistry, Queen Mary University of London, London, E1 2AB UK

**Keywords:** Process evaluation, Chronic headache, Behaviour change intervention, Fidelity, Mixed methods

## Abstract

**Background:**

The Chronic Headache Education and Self-Management Study (CHESS) multicentre randomised trial evaluated the impact a group education and self-management support intervention with a best usual care plus relaxation control for people living with chronic headache disorders (tension type headaches or chronic migraine, with or without medication overuse headache). Here we report the process evaluation exploring potential explanations for the lack of positive effects from the CHESS intervention.

**Methods:**

The CHESS trial included 736 (380 intervention: 356 control) people across the Midlands and London UK. We used a mixed methods approach. Our extensive process evaluation looked at context, reach, recruitment, dose delivered, dose received, fidelity and experiences of participating in the trial, and included participants and trial staff. We also looked for evidence in our qualitative data to investigate whether the original causal assumptions underpinning the intervention were realised.

**Results:**

The CHESS trial reached out to a large diverse population and recruited a representative sample. Few people with chronic tension type headaches without migraine were identified and recruited. The expected ‘dose‘of the intervention was delivered to participants and intervention fidelity was high. Attendance (“dose received”) fell below expectation, although 261/380 (69%) received at least at least the pre-identified minimum dose. Intervention participants generally enjoyed being in the groups but there was little evidence to support the causal assumptions underpinning the intervention were realised.

**Conclusions:**

From a process evaluation perspective despite our extensive data collection and analysis, we do not have a clear understanding of why the trial outcome was negative as the intervention was delivered as planned. However, the lack of evidence that the intervention causal assumptions brought about the planned behaviour change may provide some insight. Our data suggests only modest changes in managing headache behaviours and some disparity in how participants engaged with components of the intervention within the timeframe of the study. Moving forwards, we need a better understanding of how those who live with chronic headache can be helped to manage this disabling condition more effectively over time.

**Trial registration:**

ISRCTN79708100.

**Supplementary Information:**

The online version contains supplementary material available at 10.1186/s12883-022-02792-1.

## Background

The Chronic Headache Education and Self-Management Study (CHESS) was a multicentre randomised controlled trial comparing a group education and self-management intervention with a best usual care plus relaxation control for participants living with chronic tension type headaches, chronic migraine with or without medication overuse headache [1]. It was a large, adequately powered, trial exploring the effect of the self-management intervention on the adverse impact of headache (social functioning, role functioning, vitality, cognitive functioning, psychological distress and pain). No detectable effect was found for the intervention on headache related quality of life at 12 months. This was measured with HIT-6 (Headache Impact Test) - a measure of the adverse impact of headache on social functioning, role functioning, vitality, cognitive functioning, and psychological distress. Among the predefined secondary outcomes, self-efficacy was improved in the self-management group at four and 12-months but there were no observable effects on headache days and severity, anxiety or depression. (Ref -Monograph and main paper).

Evaluation of the principal processes of a complex study helps to explain how an intervention could be optimised or why it may have failed [2–4]. There is now a growing body of published process evaluations that help to put trial results into context [5–9]. We conducted an extensive process evaluation (PE) alongside the CHESS trial using a mixed methods design collecting data on: context, reach, recruitment, dose-delivered, dose received, fidelity, and experiences of involvement in the trial [10]. In this paper, we report this process evaluation and explore why the intervention failed to achieve a detectable clinical effect [11, 12].

### The CHESS randomised controlled trial

**Fig. 1:**
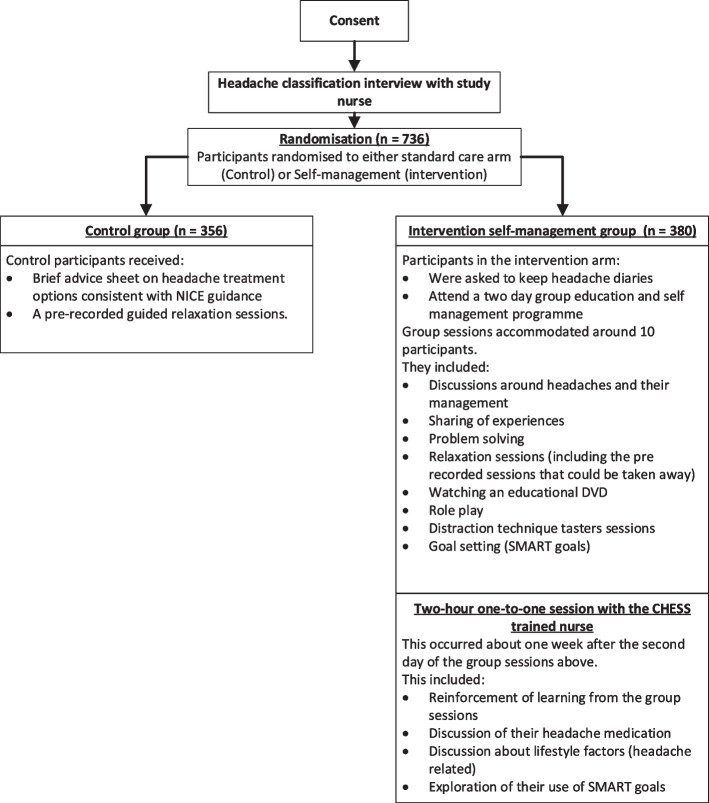
CHESS Trial participants pathway through the trial and brief outline of the interventions

This was undertaken between 2017 and 2020 in two areas of the UK (The Midlands and Greater London). We published our intervention development [13], feasibility study [14] and protocols for the trial [15] and process evaluation [10]. The latter includes the causal assumptions of the intervention. Participants were primarily recruited from 164 general practices following a search of electronic clinical records that was wide in scope as chronic headache was poorly coded. For the trial there were 736 randomised participants with chronic headache, the primary analysis was on the 727 participants with migraine. For this evaluation we include all 736 participants. The intervention used a cognitive behavioural approach including behaviour change techniques aimed at helping participants manage their headaches better. Following informed consent, but prior to randomisation, participants had a consultation with a CHESS trained nurse to classify their headache [13]. All participants and their GPs were informed of the classification of their headache. Figure 1, below, outlines the participant pathway through the CHESS trial and the basic components of the interventions.

The aims, objectives and methods used in this mixed-methods process evaluation are published in a protocol paper [10]. To aid the reader we paraphrase/briefly summarise these below.

### Aims and objectives

#### Aims

The aim of the process evaluation was to aid in the understanding of the results of trial outcome.

#### Objectives

Our specific objectives were look at implementation processes, i.e., recruitment, reach, dose delivered, dose received, delivery of the intervention and acceptability/use of the intervention in practice and fidelity. In addition, carry out qualitative work around experiences with participants and the CHESS research team.

## Methods

### Study design

The process evaluation was guided by the MRC framework and included key components of process evaluation proposed by Steckler and Linnan [3, 4]. Table 1 shows the sources and types of data for each component of the evaluation.

**Table 1:** Process evaluation (PE) components, sources, and type of data

Key PE components	Source of data	Type of data
**Context and Reach**	NHS GP practice data and trial data	Practice numbers and location. Demographic and socioeconomic characteristics of population served by the practice
**Recruitment**	Trial recruitment data	Routine trial data e.g., numbers recruited, number declined, eligible Sample of expression of interest forms from those who declined to participate
**Dose delivered**	Trial intervention data	Numbers of groups delivered/not delivered and why, location of groups
**Dose received**	Trial intervention attendance sheets Trial data	Attendance data Reasons given for not attending
**Fidelity**	Intervention group audio recordings Trial specific records of one-to-one consultations with participants	Audio recording data 10% form completion check for adherence
**Experience of participating in the trial**	Staff focus groups Participant interviews Participant feedback forms GP feedback forms	Intervention staff focus group notes and recordings Patient interview recordings / transcripts Participant feedback GP feedback

### Patient and public involvement

The CHESS reference group of 47 lay members, provided advice throughout the study [16]. They were invited to a virtual meeting held via MS TEAMS to discuss the process evaluation results.

### Data collection and primary analysis

We extracted relevant data from publicly available records and trial specific case report forms.

We distributed feedback forms to:

- participants who attended group sessions asking them about the venues, facilitators and sessions, and
- participating GP practices about their experiences of being in the trial – this was discontinued when the COVID 19 pandemic started to avoid burdening practices.

We entered quantitative data onto the study database and analysed using descriptive statistics. We took a random 3% sample of expression of interest forms from people who chose not to enter the trial and categorised reasons given. We assessed dose received based on the a priori definition of adherence for the trial: partial = day one plus one-to-one session, full = two sessions plus one-to-one.

We assessed fidelity of eight components of the intervention (see Table 4) from audio recordings of group sessions using an approach used in a previous study [17]. We took a random sample of three of these components from each group (see Supplementary file 1, Tables S1 – S3 & Fig. S1, for further detail). One researcher (VN or DE) listened to these and rated adherence (whether the intervention was delivered as directed in the facilitators’ manual) and competence (a subjective assessment of facilitation) [17]. We assessed interrater reliability through DE and VN independently rating an 11% sample and comparing results. We took a random 10% sample (*n* = 27) of the case report forms completed as part of the one-to-one sessions and assessed whether the proforma for the session was fully completed.

We interviewed participants from both arms of the trial at four and 12-months post randomisation. Recruitment to the process evaluation is detailed in the process evaluation protocol. All participants provided informed consent to participant in the trial and a purposive sample of these who, at the time of consent, agreed that they could be contacted to consider involvement in the process evaluation interview study were informed and consented to this [10]. We sampled for diversity of practice context and type of headache continuing until we achieved data saturation from participants in both trial arms. In the four-month interviews we explored experience of intervention and asked intervention participants about each of the sessions from the two days and their experience of the one-to-one that followed this. In the 12 month interviews we explored impact on headache management of the intervention. Interviews were mostly face-to-face at four-months and by telephone at 12-months. All interviews were audio recorded with consent. Interviews were transcribed verbatim and identifying data removed prior to analysis using the framework method [18].

On completion of the trial, we held focus groups with the nurses and allied health professionals (AHP) who delivered the intervention. These consisted of two parts: 1) nurses and AHP facilitators reflected on the training they received and their experiences of delivering the intervention; 2) nurse facilitators reflected on the nurse specific training they received and delivering the nurse one-to-one sessions. We analysed thematically.

We reported our full analysis in detail, online, prior to trial results becoming available [19]. In this paper, we present the results that provide insight into what participants received from the intervention and its impact. We report analysis supported by quotes labelled with participant ID number (e.g. 14).

### Post-trial result analysis

After the trial results were available, we reinterrogated our analysed data from the four-month interviews on specific intervention components to identify whether any changes intervention participants attributed to intervention components matched the causal assumptions used in intervention design (See Supplementary file 2, Fig. S4 & [10]). DE, ST, and FG independently reviewed the analysis results and compared them with all six of the causal assumptions, then compared results, discussing discrepancies to achieve consensus.

## Results

### Context and reach

We recruited from 164 general practices, with a median list size of 8979 (IQR 5760 to 11,986) including urban and rural locations (see also Supplementary file 3, Table S2). We included practices based in all ten deciles of the Index of Multiple Deprivation, median 5 (IQR 2 to 8). People from minority ethnic groups were over-represented in our practices; 27% (SD 23.4) compared to 14% in England and Wales in the 2011 census. Overall, 18% of participants recruited came from minority ethnic groups.

### Recruitment

We approached 31,020 people, from the 164 practices representing around 2% of the practices’ population. Of these 2178 (7%) expressed an interest in the study (See Table 2). We randomised 3% 736 (380 intervention: 356 control) people into the CHESS trial. In our 3% sample of responses from people saying they were not interested in joining the trial (*n* = 85), the main categories of reason given were:

- their headaches were not bad enough to make joining the group worthwhile
- attending the headache self-management programme would take up too much of their time.

**Table 2:** CHESS recruiting GP practices grouped by index of multiple deprivation deciles

*IMD	Number of practices	Patient Population of practices	Size of practices by patient population		identified at practices and contacted		interested		Interested & eligible	Eligible & consented		Eligible & randomised		
Dec	N	Tot N	Median	IQR	N	%	N	%	N	%	N	%	N	%
**1**	16	125,898	7199	(5103, 9240)	2919	2	152	5	77	51	51	66	46	60
**2**	26	217,409	8732	(6343, 10,841)	5425	3	244	5	115	47	78	68	73	63
**3**	15	160,651	7852	(7232, 12,327)	2816	2	175	5	77	44	62	81	56	73
**4**	18	140,742	6629	(5377, 10,045)	2913	2	201	7	93	46	67	72	64	69
**5**	21	194,130	9250	(5582, 11,300)	3958	2	261	7	129	49	88	68	84	65
**6**	16	154,602	10,048	(5498, 12,515)	3169	2	230	7	102	44	70	69	64	63
**7**	9	58,016	4820	(3317, 9000)	1344	2	120	9	44	37	35	80	29	66
**8**	13	123,674	9474	(6821, 12,215)	2395	2	239	10	127	53	99	78	98	77
**9**	14	172,112	12,016	(7983, 16,465)	2483	1	248	10	122	49	99	81	93	76
**10**	16	176,452	11,809	(8225, 14,084)	3598	2	308	9	148	48	106	72	99	67
**All**	**164**	**1,523,686**	**8979**	**(5760, 11,986)**	**31,020**	**2**	**2178**	**7**	**1034**	**47**	**755**	**73**	**706**^**a**^	**68**

*IMD Index of Multiple Deprivation. The deciles are calculated by ranking the 32,844 Lower-layer Super Output Area (LSOA) level in England from most deprived to least deprived and dividing them into 10 equal groups. LSOAs in decile 1 fall within the most deprived 10% of LSOAs nationally and LSOAs in decile 10 fall within the least deprived 10% of LSOAs nationally

^a^ 30 participants were self-referrals and not attached to a particular practice so are not included here

### Dose delivered

We successfully delivered 42 of 43 of the two-day group sessions in a variety of venues close to our recruiting practices (one session towards the end of the trial was cancelled due to the small number of participants who were incorporated into another group).

### Dose received

Of those randomised to the self-management intervention 288/380 (76%) attended at least one day. Of the 288, 227 (79%) attended both days and 261 (91%) took part in the one-to-one classification and advice session. Partial and full adherence was achieved by 261 (69%) and 57% (217/380) respectively. The median number of people attending on day one was 6.5 (IQR 5 to 9). Reasons for absences when provided included migraines, feeling unwell, work and family commitments.

### Intervention fidelity

#### Intervention groups

We analysed recordings from 33 of the 42 groups, analysing 90 of the 99 sessions. Reasons for missing data included technical issues and facilitators forgetting to switch on recorders. Interrater reliability was 80% for adherence scores and 90% for competence scores.

The overall adherence score was 83% (IQR 67 to 100%) with several components achieving 100%. The two components achieving the lowest scores were *‘Unhelpful thinking patterns and finding alternatives’* 75% (IQR 72, 87%) and *‘managing setbacks’* 63% (IQR 58, 77%) (See Table 4).

The overall competence score was 70% (IQR 50 to 90%) but varied across components of the intervention. The highest competence score was for delivering ‘*Acceptance of chronic headaches’* (90% (IQR 65 to 95%)) and the lowest was for *‘Impact of thoughts, and mood and emotion on headaches’* and ‘*communicating better with health professionals’* (both scored 60% (IQRs 60 to 80% and 50 to 80%, respectively)) (See Table 3).

**Table 3:** CHESS intervention Fidelity; Adherence and competence scores

Intervention Component	Adherence	Competence
	Median % (IQR)	Median % (IQR)
**Headache information and mechanisms**	89% (79, 89%)	70% (60, 80%)
**Acceptance of chronic headaches**	100% (80, 100%)	90% (65, 95%)
**Impact of thoughts, mood and emotions on headaches**	90% (60, 100%)	60% (60, 80%)
**Headache cycle and breaking the cycle**	100% (79, 100%)	80% (70, 80%)
**Unhelpful thinking patterns and finding alternatives**	75% (72, 87%)	85% (70, 90%)
**Identifying barriers to change and exploring problem solving and goal setting**	90% (70, 98%)	80% (75, 100%)
**Communicating better with healthcare professionals**	100% (86, 100%)	60% (50, 80%)
**Managing setbacks**	63% (58, 77%)	75% (68, 83%)
**Overall**	**83% (67, 100%)**	**70% (50, 90%)**

#### Fidelity check of Nurse-led one-to-one sessions

Proformas were all fully completed as required by the trial protocol [19].

### Participants’ experiences of CHESS intervention

The interview sample is summarised in Table 4.

**Table 4:** Interview sample

	4 ms *n* = 28	12 ms *n* = 23
Control	**9**	**7**
Intention to treat^a^	**2**	**1**
Intervention	**17**	**15**

^a^Intention to treat participants are those who were randomised to the intervention but did not take part in any intervention activities

The following sections represent summaries of the full dataset which is available and where appropriate provides exemplar quotations [19].

#### Participants’ experiences of the group sessions

We identified the following five themes:

1. Discussion and sharing (being able to talk about headaches and share tips)
2. Shared experience of headache
3. Comparing with others (with some noting they were not as bad as others which made them feel better)
4. Feeling less isolated (realisation that they were not the only person who has to live with chronic headache)
5. Personal relevance (whilst most liked the groups some felt they were not relevant to them)

#### Participants comments on the group venue

Only ten participants commented on the venue in summary comments fell into the following categories:

- The venue was fine
- Issues related to personal comfort (e.g. heating, ventilation, lighting, external noise, and cramped space)
- Issues specific to the venue (e.g. car parking, and poor signage)

#### Facilitation

Fourteen participants commented on how the facilitators delivered the course. The majority felt the groups were well run and were positive about the course delivery.

*… the two people that were running it were great and they were very accommodating for us because if we went off track, they were happy to let us just explore what … we were talking about…31*

Some commented on the relaxed nature of the groups.

*… the ladies who lead the course were very good… I think we all felt very relaxed and easy… you know easy to chat…27*

Three participants wanted more expert input into the group one commenting that the facilitators were not experts in headache. Three felt the pace was slow, two felt they did not get along with one of their facilitators and two felt that one of their facilitators had delivered some sessions poorly.

*… the way it was executed by the person who was doing the facilitation was a bit muddled up so we didn’t fully understand what we were supposed to be doing… and then she did spend a whole couple of minutes literally reading… through the slides 25*

#### Intervention sessions

Table 5a and b summarise responses of participants when asked about the specific intervention sessions. Overall, the sessions were acceptable, but some sessions were liked more than others and some sessions were felt to be irrelevant for some people.

**Table 5:** Participant responses to being asked about their experience of and impact from specific group sessions

**Day 1 Living, understanding, and dealing with chronic headaches**	
Session 1&2. Welcome & Introduction	No specific comments on these
Session 3*. Headache information and mechanisms *n* = 14	7 gained new information about characteristics and classification of different headaches 7 were previously aware of medication overuse headache (MOH). Of these, 2 thought it relevant to them but were resistant to decreasing medication and 1 thought it not relevant for them 7 were unaware of MOH and were either surprised or found it counter intuitive. Of these, 4 decided to change their medications, 1 came off their medication altogether, 1 was resistant to decreasing their medication, 2 thought it not relevant for them
Session 4*. Acceptance of chronic headaches *n* = 12	10 participants found it useful and relevant to living with headaches, *“...helps you to think slightly differently about things.” 23* 6 participants recognised where they were on the acceptance curve: “*That’s kind of been me!.....that has stuck with me…“15* “*I’m past the phase of ‘Why me?”‘09, “I’m definitely at the acceptance stage…“23* 3 didn’t find this session useful “*I found that [acceptance session] bizarre.”24*
Relaxation and breathing *n* = 13	5 had not used the CD or relaxation after the course 3 used the CD and were continuing to use some form of relaxation. 2 used their own established form of relaxation 3 had no time to fit relaxation into their lives 2 thought they did not need relaxation as they were not stressed
Session 5. Impact of thoughts, mood and emotions on headaches *n* = 11	11 agreed there was a strong link between mood and headache of which 1 did not understand session aim and content as found delivery unclear
Session 6*. Headache cycle and breaking the cycle *n* = 10	6 thought it useful to look at headaches in a different way 3 thought headache cycle was easier said than done 3 did not feel it was personally relevant
Session 7*. Unhelpful thinking patterns: recognising and finding alternatives *n* = 12	5 identified with unhelpful thinking, *“… we were able to identify things that we were doing and everyone was going ‘oh yeah yeah’…“31* 5 changed their thinking to be more positive using the reframing techniques taught *“that was quite interesting actually it was like actually ‘turn your thoughts around and think well what can I do……is there anything I can do to help myself?’ So that was really good.” 30* 3 had heard about the technique before of which 1 did not get on with it 4 did not find it useful, 1 said it had not been explained well enough 1 decided to see their GP about antidepressants due to this session.
Educational DVD *n* = 15	11 had watched the DVD of which four watched with someone else (a relative or friend) and found this useful. Of those who had watched it, 5 found it personally useful, 4 already knew the content and 2 had no memory of the content 4 had not watched the DVD of which 2 had no way to play it
**Day 2 Learning how to adapt and take control of your life with chronic headaches**	
Session 10*. Identifying barriers to change and exploring problem solving and goal setting *n* = 12	4 already knew about goal setting 7 found goal setting useful: increasing their fluid intake (2) decreasing or changing their medication (2) doing mindfulness (1) or increasing their practice, (1) and improving their bedtime routine to help their sleep quality (1). Of these 3 had achieved their goals, 3 had not and for 1 it was work in progress 5 found goal setting was ‘not for them’ (2), difficult (2) or provoked anxiety (1)
Session 11. Lifestyle factors and impact on headaches *n* = 13	All found this useful *“…to understand what it is you are actually doing and see if there is a link and a connection to the headaches...” 09*
Session 12*. Managing stress and anxiety *n* = 10	All acknowledged link between stress and headaches but for most doing something about it was difficult 3 had considered making or made changes. *“My job is a very stressful … when I asked they did reduce some of the job for me…… even just going for a walk it can help you reduce it [stress] but before I didn’t really know that.” 24*
Session 13. Managing sleep better *n* = 10	4 said they slept well and 2 found the information helpful. 4 were already aware of the information and for one shift work was a problem, “*It wasn’t anything that I didn’t know already … it is very, very difficult because of shift work…” 31*
Session 14*. Mindfulness and relaxation for headaches *n* = 17	3 were using mindfulness successfully: 1 already practised it, 1 restarted it and 1 commenced it 3 used mindfulness informally *“But you see if I pick that cross stitching up I can’t think of anything else but that while I’m doing it so my mind’s completely blank from anything else and I think that helps me.”28* 8 found it not personally useful: 4 because focusing increased other symptoms of stress, pain or headache; 4 did not understand what mindfulness was and 4 had no time in their lives to try it
Session 15*. Medication management *n* = 10	9 liked hearing about the different medications available for migraine. *“I mean I’ve had migraines for years and no one’s ever suggested this before” 31* of which 2 used the information to discuss medication with their doctor*.* 2 wanted more in-depth personally tailored information
Session 16. Relationships and communication with family, carers and friends *n* = 4	3 found the listening exercise enjoyable 1 found it irrelevant
Session 17*. Communicating better with Health Professionals *n* = 9	4 found the role play helpful: “*When I was first going in [to doctors] with my headaches it was like, ‘Well just keep taking Paracetamol’, ‘Well it’s not doing anything’ … so it was quite good to actually go in and be like ‘right ok doctor I want to be put on a preventative I can’t live with my headaches like this’… really helpful.” 30* 4 did not find it personally useful 1 thought it not useful “*if they’re [doctor] not gonna listen you are not gonna get anywhere… a lot of times they need to learn their bedside manner”17*
Session 18. Managing setbacks – what to do when things don’t go to plan *n* = 7	All 7 remembered little about the session except it rounded off the course and that setbacks are part of life.

*n* number of participants who comment on session from among the 17 interviewed at 4 months

*Some participants made more than one comment about each session

#### Nurse led one-to-one sessions

Participants generally welcomed these one-to-one sessions. They liked being able to talk about their headaches with some who would listen. Headache classifications that were provided during these sessions were appreciated by most, but they also brought about a confusion, misunderstandings, and some disbelief in the classifications given. There was little or no evidence that goals had been set or achieved by participants.

#### Diaries

Liked about headache diaries:

- Able to monitor changes
- Reassuring (especially if seeing improvements)
- Helped identify triggers or patterns (or none)
- A good aid when talking to the GP about their headaches

Disliked about headache diaries:

- Being reminded about their condition
- Difficult to complete regularly
- Paper would prefer an App

#### Telephone support

Some noted that they felt they didn’t need this additional support, so they did not use them.

Others commented on what they liked about the telephone support.

Liked:

- Useful to reflect on how they were doing
- Appreciated the additional support

#### Changes attributed to participating in the intervention

Fifteen group intervention interview participants contributed to this data from the 12-month interviews. We identified seven themes (see Table 6). Participants often gave responses in multiple themes (See Supplementary file 4, Table S3 for more details).

**Table 6:** Changes in how participants managed their headaches

Doing things differently: Seven said there was a change in their headache management which included lifestyle factors or reinforcing good practice such as being hydrated, taking breaks, having regular meals, doing relaxation to help with mood, mindfulness or applying pacing strategies. One spoke about getting additional help to try to change their unhelpful thinking habits.	
It makes you think: Four said that the group had ‘made them think’ allowing them a time of reflection.	
New knowledge: Three felt that they had acquired new knowledge about medication overuse or headache triggers.	
Changes in medication: Three people had changed their medication, two with an added preventative giving a decrease in headaches and one by adding a triptan which helped give them the flexibility and management of severe headaches.	
Change in attitude: Two spoke about a change in their attitude towards their headaches which had given them more freedom socially and some had taken on new activities. In order to make changes this person needed to address their depression first.	
Raising research awareness: Four were appreciative of the research in raising the awareness of chronic headaches.	
No change: Five reported no change in their management or knowledge of headaches after attending the intervention either because they felt they knew it already or because their headaches didn’t interfere with their lives or that it wasn’t personally relevant.	

### Participants’ experiences of control intervention

Among the nine control intervention participants interviewed, several found the pre-recorded guided relaxation session useful and used it regularly. Others did not find time in the day to devote time to relaxation or found the CD format inconvenient. In terms of the information provided by the study team, four control participants could not recall getting the information. Of the rest: One noted that the information was nothing new to them but then said that they did like the information about medications. Another noted that the information provided made them think about the triggers for their headaches. One participant commented that it made them aware of medication overuse headaches and as a result was careful with taking over the counter medications, also commenting that the information generally was useful. The final participant appreciated the information provided and being part of the study saying that the study “validated their headaches…”.

### Facilitator experiences explored in focus groups

Twelve nurse facilitators and four allied health professional facilitators participated. The facilitators were generally happy with their training. They noted that a considerable amount of unplanned preparation time was needed before each session. There were sometimes long delays between training and session delivery and refresher training would have been beneficial. Facilitators found some sessions more challenging to deliver, notably: Session 4. Acceptance, Session 5 Impact of thoughts mood and emotions on headaches, Session 14. Mindfulness and relaxation for headaches, Session 15. Medication management, and Session 18. Managing setbacks.

Nurse facilitators found their specific training excellent, although more role play might have been beneficial. They found the CHESS manual very useful. The one-to-one sessions required more time than planned with many participants divulging much personal information. The nurse facilitators suggested that some debriefing would have been helpful. They found MOH was a difficult topic although they felt that some participants came to a realisation of its relevance. They noted that most participants reported cutting down their medication gradually with good results [19].

### Feedback forms from intervention participants

The feedback form was completed by 117 participants at the end of the two-day group session. In general feedback was positive. Most valued was meeting and sharing with others. Least valued included the mindfulness and relaxation sessions (See Supplementary file 5, Table S4 for more details).

### General practitioner feedback

We collected data from 25/64 practices. Responses to the three questions were:

1. What were your experiences of treating patients with chronic headache within the CHESS trial? 12 positive, 10 neutral, 0 negative, 3 no response
2. To what extent, if at all, has the CHESS trial changed your approach to treating chronic headache? 11 positive, 12 neutral, 0 negative, 4 no response
3. How would you describe your practice’s involvement in the CHESS trial, and would you be happy to be involved in similar trials in the future? 23 positive, 1 neutral, 0 negative, 1 no response

### CHESS reference group discussion

Ten lay members attended the group discussion. Whilst they were disappointed generally with the results, they were also not that surprised. Noting that whilst this study tried a novel approach to the treatment of chronic headache, they felt that the results implied it was clearly not enough. They felt that chronic headache is under researched, a ‘hidden’ condition, which is often misunderstood and a major cause of disability and time off work, impacting on all aspects of life. The group noted that chronic headaches should be seen as a long-term condition which needs coordinated long term medical review, support, signposting to appropriate clinicians, information and services which may be of benefit. Future research will be important to explore the most useful avenues. The group felt that the results of this study should be presented in a way that gives clinicians and the public an understanding that chronic headache is a complex condition which should be taken seriously and that there is further work to be done.

### Post-trial analysis

We found little evidence that the changes that intervention participants attributed to intervention components matched the causal assumptions of the intervention design. For three components where there was evidence, we found there was an equal about of contrary evidence. See Table 7.

**Table 7:** The extent to which the causal assumptions of the intervention were met

	Causal assumption	Synthesis of findings
1	Increasing understanding and acknowledging unhelpful beliefs and behaviours about their headaches	Only a small proportion of participants increased their understanding and acknowledged their unhelpful beliefs.
2	Learning and applying techniques for managing chronic headaches	Some evidence is found within the data that some participants were making changes in line with this assumption. There was an equal amount of data to the contrary.
3	Encouraging re-activation and re-engagement to improve quality of life	There was very little evidence in the data to suggest that this happened in the group sessions or after the end of the study
4	Promoting individual independence	There was little evidence in the data to suggest that this happened in the group sessions. There were a few examples after 12 months.
5	Using facilitators to guide participants to discover and generate new ideas, beliefs and behaviours via a group learning process.	Some evidence is found within the data that some participants were making changes in line with this assumption. There was an equal amount of data to the contrary.
6	Having one to one engagement to discuss medication management and offering ongoing support thereafter	Some evidence is found within the data that some participants were making changes in line with this assumption. There was an equal amount of data to the contrary.

## Discussion

The CHESS intervention was delivered as planned with good levels of fidelity consistent with similar interventions [17]. Over two thirds of participants received at least the planned minimum dose, consistent with other chronic disease self-management studies [20].

The opportunity to meet and have discussions with others who had a similar condition was valued by almost every study participant interviewed. Intervention group sessions were perceived as informative, but few people reported making changes to how they managed their headaches because of specific content. Most study participants had previous experience of keeping a headache diary. Although most participants valued the opportunity to discuss their medication in the one-to-one session with a nurse, only a few went on to discuss their medication with their GPs. Few participants interviewed for the process evaluation spoke of goal setting and none reported having achieved their goals. Twelve months after the intervention, participants reported little change to their headache management. There was little evidence to suggest that the causal assumptions for our intervention were realised within the time frame of our study.

Chronic headache is a condition that becomes embedded in all aspects of life. Our own systematic review completed in the early stages of this study, found that headache is a driver of behaviour, affects people’s relationships and is an ever present ‘spectre’ over their lives [21]. This is echoed in more recent studies. A survey from Norway indicates that living with a chronic headache condition is associated with high levels of disability and considerable negative consequences for daily living [22]. A US survey which included over a 1000 people with chronic migraine demonstrates the negative impacts that the condition has on sufferers including relationships, careers, finances, achievements, and overall health [23]. An interview study from Spain found pain becomes the main focus of life and strongly impacts work and family [24]. Our intervention was insufficient to prompt enough change in the way our participants managed their headaches and their lives to bring about a detectable effect for the intervention on headache related quality of life at 12 months. There is emerging evidence that suggests why this might be. An interview study exploring goal management in chronic headache demonstrates the complexities of goal management, the effort involved and that this can be a prolonged process sometimes involving major life changes such as changing work or avoiding expanding family size [25]. Some of our participants reported modest goal setting such as monitoring fluid intake and improving sleep hygiene with a few considering or making major changes to their life such as their work within the time frame of our study. A qualitative study of people with chronic migraine and MOH explores the difference between those who frequently relapse after structured withdrawal for MOH and those who less frequently relapse. The former tend to attribute headache to uncontrollable factors, are resigned to their headaches and use passive coping strategies [26]. This may help explain the range of responses to the component of our intervention on MOH. Studies such as these that explore the complexities of responses to components of our intervention may help us understand how to personalise interventions both in terms of content and duration. Such interventions may benefit from a multidisciplinary approach [27].

### Strengths and weaknesses of the evaluation

Our process evaluation was extensive and followed a published protocol. Our assessment of the fidelity of the intervention was comprehensive. The interview sample may not be large enough to capture the diversity of ways participants responded to the intervention. GP feedback was limited due to the COVID pandemic. Evaluation of the one-to-one sessions was limited to clinical record forms. Nearly one third of those in the intervention arm did not attend enough to achieve our definition of “partial adherence”, this could, potentially, have diluted the effects of the intervention. Finally, whilst we approached a large population the trial failed to identify and recruit those with TTH without co-existent migraine and as a result was predominantly those with migraine.

## Conclusion

Our intervention for chronic headache was delivered as planned and received by two thirds of participants. However, within the time frame of the study, there were only modest changes in how participants managed their headaches. The variation in how people responded to the components of the intervention suggests we need more understanding of how and why people respond as they do.

## Supplementary Information

**Additional file 1: Supplementary file 1.** Group session intervention fidelity

**Additional file 2: Supplementary file 2.** CHESS Process evaluation logic model

**Additional file 3: Supplementary file 3.** Areas in the UK where CHESS groups were delivered

**Additional file 4: Supplementary file 4.** Changes attributed to participating in the intervention arm of CHESS reported after the 12 month questionnaire.

**Additional file 5: Supplementary file 5.** Participant feedback (forms completed after the 2-day sessions)

## Data Availability

Much of the data generated or analysed during this study are included in this published article [and its additional information files and published work]. Reasonable requests for the datasets used and/or analysed during the current study can be requested via the corresponding author.

## Abbreviations

CHESS: Chronic Headache Education and Self-Management Study
MOH: Medication overuse headache
TTH: Tension type headache
NHS: National Health Service
PE: Process evaluation
PPI: Patient and public involvement
MS TEAMS: Microsoft Teams software
GP: General practitioner
IQR: Interquartile range
