# Supplementary material for: Chronic Headache Education and Self-Management Study (CHESS): a process evaluation
Source: BMC Neurol. 2023 Jan 7;23:8. doi: 10.1186/s12883-022-02792-1 (PMC9823254; doi:10.1186/s12883-022-02792-1)
Supplement: Supplementary file 1 — Additional file 1: Supplementary file 1. Group session intervention fidelity [file 12883_2022_2792_MOESM1_ESM.docx]

Supplementary file 1: Group session intervention fidelity

Here we provide further information on the fidelity measurement in the CHESS Process Evaluation.

Our methodology is based on previous work*. Mars T, Ellard D, Carnes D, Homer K, Underwood M, Taylor SJ. Fidelity in complex behaviour change interventions: a standardised approach to evaluate intervention integrity. BMJ open. 2013;3(11).*

Key components were identified, before the trial commenced, which were considered by the team to be ones of education or were underpinned by theories which promoted behavioural change and/or self-management. These were in contrast to sessions which were; introductory, summarising, carrying out practical strategies such as relaxation, mindfulness or those giving general lifestyle advice including stress and sleep. Eight sessions were identified: Day 1: 3,4,5,6,7 Day 2 10,17,18. See details in Table S1 below.

Table S1. Key Sessions for fidelity Aims/Rationale

| **Day 1 Living, understanding and dealing with chronic headaches** | | |
| --- | --- | --- |
| Sessions | Aim | Rationale |
| ***Session 3 50 mins**  Headache information and mechanisms | To increase understanding of chronic headache and reasons for it | Information and education to increase awareness |
| **Break** | | |
| ***Session 4 45 mins**  Acceptance of chronic headaches | To introduce the concept of acceptance and need for self-management | Principles of acceptance therapy |
| **Lunch** | | |
| ***Session 5 30 mins**  Impact of thoughts, mood and emotions on headaches | To start to introduce the concept that pain and mood are linked and that mood can have an influence on headaches | Understanding emotional consequence |
| ***Session 6 30 mins**  Headache cycle and breaking the cycle | To explain the pain cycle individuals can get stuck in due to the unhelpful things we do and explore the strategies that can be used to help break the cycle that keep us in that cycle | Education and shaping knowledge, based on Vlaeyen’s Fear avoidance model |
| **Break** | | |
| ***Session 7** **40 mins** Unhelpful thinking patterns and finding alternatives | To introduce ideas about unhelpful thoughts, automatic thoughts and errors in thinking. To understand the impact of unhelpful thinking and how such thought patterns can keep people in the pain cycle and explore ways to reframe these thoughts | Challenging unhelpful thought patterns (based on the fundamentals of Rational Emotive Therapy, CBT techniques and change management principles) |
| **Day 2 Learning how to adapt and take control of your life with chronic headaches** | | |
| **Sessions** | **Aim** | **Rationale** |
| ***Session 10 30 mins**  Identifying barriers to change and exploring problem solving and goal setting | To get participants to think about future goals and explore these by identifying possible barriers, potential solutions and develop an associated action plan | Goals and planning, theory of planned behaviour, theory of reasoned action based on CBT principles |
| **Break** | | |
| ***Session 17 20 mins**  Communicating better with healthcare professionals | To reflect on consulting behaviour and promote effective communication and constructive consultations | To promote effective healthcare utilisation |
| **Break** | | |
| ***Session 18 25 mins**  Managing setbacks- what to do when things don’t go to plan | To know what to do when experiencing a setback or a flare up | Preparation and embedded learning |

It was planned that all two day group sessions would be audio recorded it was these audio recordings that would be used to analysis sessions. The audio recordings of a random selection of 3 of the sessions per group were scored for adherence and competence by one researcher. There was a 10% check of these by a second researcher to assess inter-rater reliability. Our a priori sampling frame is shown in figure S1 below. Four from the eight sessions were randomly selected for each group using excel random number function. The fourth session randomised was deemed as a backup in the event a session was not recorded (missing).

Figure S1. Sampling grid for CHESS group session fidelity

Analysis of recordings did not start until all sessions had been delivered. The researchers gathered together the recordings and for each group listened to and evaluated the specific sessions identified on the sampling grid.

The aim was to evaluate:

- Adherence (delivery of the intervention as per protocol)
- Competence (“how well was the session facilitated?”)

Figures S2 and S3 below are examples of the scoring sheets – for adherence these were session specific and mapped against the facilitator’s manual.

The researchers listened to the sessions and looking for items to be ‘evident’; they rated this for each item as either, Yes (2 points) Partially (1 point) No (0 points) – scores are totalled and a percentage generated for both adherence and competence.

Figure S2. Example CHESS Fidelity Adherence score sheet (Day 1 / Session 6/ Title: Headache cycle and breaking the cycle – 30mins)

| No. | Item | Adherence | Comments |  |
| --- | --- | --- | --- | --- |
| 6.1 | Did the facilitator(s) show the group the persistent headache pain cycle, and explain the vicious circle? | Yes (2)  Partially (1)  No (0) |  |  |
| 6.2 | Did the facilitator(s) ask the group to generate a list of unhelpful things that people might do that keep them in the cycle? | Yes (2)  Partially (1)  No (0) |  |  |
| 6.3 | Did the facilitator(s) give the group the depressive symptom list (handout 1) and ask them to consider these at home? | Yes (2)  Partially (1)  No (0) |  |  |
| 6.4 | Did the facilitator(s) suggest that if they are worried about these symptoms overwhelming their lives that they should consider seeing their GP? | Yes (2)  Partially (1)  No (0) |  |  |
| 6.5 | Did the facilitator(s) ask the group what they could do to break the cycle? | Yes (2)  Partially (1)  No (0) |  |  |
| 6.6 | Did the facilitator(s) show or talk about the pain cycle diagram with escape routes? | Yes (2)  Partially (1)  No (0) |  |  |
|  | Total adherence score __/12 |  |  |  |
|  | Percentage adherence score (Total adherence score __/12 x 100) |  |  |  |
| **Instructions:**  When at all possible please rate as ‘Yes’ or ‘No’ If ‘partially’ then write reason in comments box  Questions need not be verbatim (unless specified) as long as content of session is covered.  **Comment** | | | | |

Figure S3. Example CHESS Fidelity Competence score sheet

| **CHESS** | Item | Competence measure | Comments |
| --- | --- | --- | --- |
| Introduction | Did the facilitator(s) ‘set the scene for the session? *e.g. did they introduce the title, aims or content of the session?* | Evident (2)  Partially evident (1)  Not evident (0) |  |
| Discussion | Did the facilitator(s) encourage individual and group participation? *e.g. did they; encourage individuals to participate, ask open questions, give enough time for the group to answer (rather than answer their own questions)* | Evident (2)  Partially evident (1)  Not evident (0) |  |
| Group climate | Did the facilitator(s) foster a positive group climate? *e.g. Did they say positive things about people ‘that’s a helpful comment’ ’thank you for sharing that’. Did they encourage sharing and exploration of ideas in a non-judgemental way?* | Evident (2)  Partially evident (1)  Not evident (0) |  |
| Summary | Did the facilitator(s) consolidate/embed the group’s learning at the end of the session? | Evident (2)  Partially evident (1)  Not evident (0) |  |
| Linking | Did the facilitator(s) link any of the session to other sessions? | Evident (2)  Partially evident (1)  Not evident (0) |  |
|  | Total competence score __/10 |  |  |
|  | Percentage competence score (Total competence score __/10 x100) |  |  |
| **Comments:** For use if sessions; go off track, include items which are not on checklist, contain surprising unforeseen aspects or the item wasn’t covered as intended. | | | |
