# Supplementary material for: Chronic Headache Education and Self-Management Study (CHESS): a process evaluation
Source: BMC Neurol. 2023 Jan 7;23:8. doi: 10.1186/s12883-022-02792-1 (PMC9823254; doi:10.1186/s12883-022-02792-1)

Supplementary file 2: CHESS Process evaluation logic model

Figure S4. CHESS Process evaluation logic model


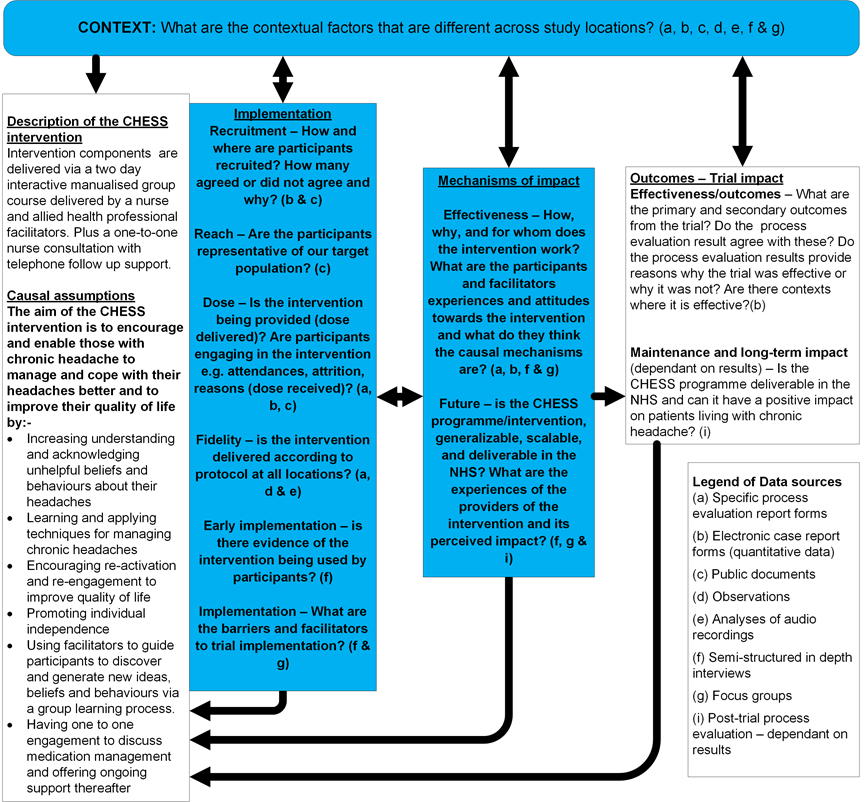

Supplement: Supplementary file 2 — Additional file 2: Supplementary file 2. CHESS Process evaluation logic model [file 12883_2022_2792_MOESM2_ESM.docx]
