# Supplementary material for: Chronic Headache Education and Self-Management Study (CHESS): a process evaluation
Source: BMC Neurol. 2023 Jan 7;23:8. doi: 10.1186/s12883-022-02792-1 (PMC9823254; doi:10.1186/s12883-022-02792-1)
Supplement: Supplementary file 3 — Additional file 3: Supplementary file 3. Areas in the UK where CHESS groups were delivered [file 12883_2022_2792_MOESM3_ESM.docx]

Supplementary file 3: Areas in the UK where CHESS groups were delivered.

Table S2. Group locations

| Locations and number of groups | | Locations and number of groups | |
| --- | --- | --- | --- |
| Bedworth - Midlands | 1 | Nottingham - Midlands | 2 |
| Birmingham - Midlands | 3 | Nuneaton - Midlands | 1 |
| Bromley - South London | 1 | Solihull - Midlands | 1 |
| Camden - North London | 2 | Southwark - South London | 3 |
| Coventry - Midlands | 3 | Stratford upon Avon - Midlands | 1 |
| Hereford - Midlands | 1 | Tower Hamlets - North London | 2 |
| Hounslow - London | 1 | Tutbury - Midlands | 2 |
| Kenilworth - Midlands | 1 | Abingdon – Thames Valley | 2 |
| Lambeth - South London | 1 | Wandsworth - South London | 1 |
| Leicester - Midlands | 1 | Wantage – Thames Valley | 1 |
| Lichfield - Midlands | 2 | Warwick - Midlands | 3 |
| Milton Keynes – Thames Valley | 1 | Witney – Thames Valley | 1 |
| Newham - North London | 1 | Worcester - Midlands | 3 |
