# Supplementary material for: Chronic Headache Education and Self-Management Study (CHESS): a process evaluation
Source: BMC Neurol. 2023 Jan 7;23:8. doi: 10.1186/s12883-022-02792-1 (PMC9823254; doi:10.1186/s12883-022-02792-1)
Supplement: Supplementary file 4 — Additional file 4: Supplementary file 4. Changes attributed to participating in the intervention arm of CHESS reported after the 12 month questionnaire. [file 12883_2022_2792_MOESM4_ESM.docx]

Supplementary file 4: Changes attributed to participating in the intervention arm of CHESS reported after the 12 month questionnaire.

15 participants contributed to this data. Analysis revealed seven themes of change attributed to the CHESS study: Doing things differently, It makes you think, New Knowledge, Changes in medication, Change in attitude, Raising research awareness and No change. Participants often gave responses in multiple themes.

Table S3. Themes of change attributed to the CHESS study

| **New Knowledge:** | Three felt they had acquired new knowledge about medication overuse (2) or headache triggers (2)  *… as I said I used to take a lot of medicine for it……but yeah when I had the study I realised it’s not all that good… it’s not good for me to be taking that much medicine so I reduced them.26*  *Yeah the course taught me how to identify what was my triggers… so I also make sure I drink plenty and I also make sure I eat regular meals as well.28*  *… I understand them more and how to cope with them so thank you very much for letting me come on the study to understand that! 28* |
| --- | --- |
| **Changes in medication:** | Three people had changed their medications two with an added preventative giving a decrease in headaches and one by adding a triptan which helped give them some flexibility with the management of severe headaches.  *…this year is… I only have eight episodes of migraine the year to date which if I compare to last year at the same period 21 and the year before 32 so… mmm… the whole of February I didn’t have any episodes and March only one… mmm… and there were some in April and so far none in May so… mmm… yeah it’s… that’s quite a transformation actually. …when I saw the doctor and talked about the programme that I’d been on and said ‘look here’s some other options of medication that were made know to me’ etc, etc and she looked at them … …and said ‘we ought to try the Candesartan’ only 8mg tablets… mmm… one a day… err… which is what I’ve been on religiously ever since … …and it’s all I can think of that… mmm… you know… that has been responsible for you know the success or the reduction in… in episodes because nothing else has changed nothing either in my life style or… mmm… diet ….09*  *… well I think it wasn’t until you guys kind of you know until I signed up to the CHESS study because I would to go the GP and they’d say ‘oh just keep taking Paracetamol’ whereas at least I had kind of a backing from… from you guys to say ‘actually you’ve got to give me something more than that’ and you put in ideas like the Propanolol’s and the fact that there were preventatives for headaches which I didn’t know about so that was yeah really helpful.30*  *… but I found it very useful you know especially the sessions if it hadn’t been for the CHESS I wouldn’t’ve known about these new tablets[sumatriptan] and those have been I feel very helpful….… it gives me more flexibility if you know what I mean because I know it’s gonna to be pretty ok after about an hour and then I can go out and if it delays it until the following day I prefer to do that cos you know I can enjoy when I’m going out… so it is… they are better and certainly more manageable and it makes it more flexible if you know what I mean. 19* |
| **Change in attitude:** | Two spoke about a change in their attitude towards their headaches which had given them more freedom socially and some had taken on new activities.  *… I used to think when I went out for a meal with the family and I didn’t feel that great afterwards… you know and we had to… you know kind of a rushed finished I used to think, ‘Oh that’s my meal spoilt’ but now I think, ‘At least… at least I went and enjoyed two thirds of it!’and also I have changed my attitude and actually it was… I might’ve got there in the end but that course speeded it up and made me start re-thinking my attitude towards headaches which is really useful…… I think the difference is more towards my attitude towards them rather than the headaches themselves there is a bigger difference in that I try and… I try and not make the assumption that I can’t do something because of my headaches but the other positive thing that I’ve done more recently is that I’ve joined a gym and now my assumption was I can’t go swimming because of the reflection on the water and I can’t do the gym because the lights will be bad…15*  In order to make changes this person needed to address their depression first.  *… I’ve kind of changed my attitude I think that’s because I feel a bit more positive taking the new medication [for depression] as before it was too much effort to try a new thing… …… the medication change has made me have the energy to try a few more things and then that has the knock on effect of making you feel a bit more positive cos you are able to do a few more things and that’s quite good.15*  *I don’t let them control me now like I used to I control them… so I don’t seem to lose out much on the social side anymore! … well normally I like lose like two to three days now I’m only losing like a day. 28* |
| **Raising research awareness:** | Four were appreciative of the research in raising the awareness of chronic headaches.  *I am pleased to know that it is being taken seriously within a doctor’s kind of environment you know it’s not just like… whereas before I could’ve probably easily felt like doctors just wanted to give you painkillers and get rid of you… I don’t quite feel so strongly that they will do that now do you know I do feel like there more being looked into than what we actually give credit for in the background so… it… it is definitely something I do not regret doing… mmm… and if ever in the future I was told that there may be some more options available to try then I would definitely be interested in finding out more about that. 23*  *you made us feel that we’re being listened to and understood and that you know people are taking it seriously and it makes all the difference makes us feel like you know we are being listening to and it is being understood that something is majorly wrong… makes all the difference I think that’s the major thing is that we feel that people are understanding that the pain that we’re in! 28*  *… and obviously helpful to realise that there are somethings that I was doing that could’ve potentially been making it worse so yes it was… it was helpful… … taking the Paracetamol… mmm… as a sort of… taking it anyway… taking it too often… well not too often but… mmm… taking it every day… …yeah so I try not to do that now because I remember them saying about the… mmm… medication overuse and I thought that was fascinating so… mmm… I’ve… err… not been doing that as often and also… mmm… that’s help me advise other people as well…31* |
| **No change:** | Five reported no change in their management or knowledge of headaches after attending the intervention either because they felt they knew it all already or because their headaches didn’t interfere with their lives or that it wasn’t personally relevant  *… some of it I felt was irrelevant and I couldn’t… I failed to see how this… mmm… related to what we were talking about…17*  *… so I quite like the group session in that respect… mmm… but I didn’t pick up anything from the… mmm… sort of tutorials if you like because to be honest I’ve been suffering with them for so long that I knew most of it… mmm… because there’s nothing new… there didn’t seem to be anything new… mmm… nobody was telling me anything I didn’t already know which was a shame cos I thought I might learn something new which… mmm… but no so… but the rest… I thought it was very good and I would still do and go for something else…24*  *… mine aren’t that bad I mean they’re not very pleasant but they don’t stop me doing things… mainly because the Sumatriptan does work generally it certainly takes the edge off the pain…27*  *… because of the fact that they didn’t really benefit me in anyway or I didn’t feel like they did I’ve not pursued… continued with them … because as I mentioned it was helpful to understand other people’s issues and to understand other ways of coping and at the same time…23* |
