# Supplementary material for: Chronic Headache Education and Self-Management Study (CHESS): a process evaluation
Source: BMC Neurol. 2023 Jan 7;23:8. doi: 10.1186/s12883-022-02792-1 (PMC9823254; doi:10.1186/s12883-022-02792-1)
Supplement: Supplementary file 5 — Additional file 5: Supplementary file 5. Participant feedback (forms completed after the 2-day sessions) [file 12883_2022_2792_MOESM5_ESM.docx]

Supplementary file 5: Participant feedback (forms completed after the 2-day sessions)

117 participants completed most of questions 1 to 8 and the results are given in Table 1 below.

Feedback forms were given to intervention participants at the end of Day 2 with a stamped addressed envelope for them to return them to the WCTU.

Quantitative satisfaction questions ‘*Please use the following scale to indicate your level of satisfaction for the following questions where 0 indicates least satisfaction, and 5 indicates most satisfaction’*.

Table S4. Results from CHESS feedback form satisfaction questions.

|  | 0  Least satisfaction | 1 | 2 | 3 | 4 | 5  Most satisfaction | Total responses |
| --- | --- | --- | --- | --- | --- | --- | --- |
| 1 The course overall |  |  | 1 | 11 | 55 | 50 | 117 |
| 2 The facilitators leading the course |  |  |  | 7 | 39 | 71 | 117 |
| 3 The group discussion process |  |  |  | 8 | 41 | 67 | 116 |
| 4 The amount of time spent on each topic |  | 2 | 1 | 17 | 42 | 55 | 117 |
| 5 The relaxation taster session |  | 3 | 6 | 17 | 36 | 55 | 117 |
| 6 The mindfulness taster session |  | 2 | 8 | 18 | 36 | 50 | 94 |
| 7 The handouts |  |  | 1 | 8 | 40 | 68 | 117 |
| 8 The course venue | 1 | 6 | 2 | 16 | 35 | 56 | 116 |

In their responses to the last three questions some gave multiple responses often about different aspects of the group intervention.

**Question 9** **What parts of the two-day course, if any, did you enjoy or value the most?**

114 participants responded we report the main themes and most cited aspects in each theme (two or less comments in each theme aren’t reported). There were three main themes:

- *Meeting, sharing and discussion with people who experienced the same condition*
  - (N=78). Some enjoyed the support and empathy of the group and others the opportunity to swap their stories as well as their management strategies.

*“The group discussions because it was very helpful talking to other people about their experiences and pick up tips from them also it helps to learn you aren’t the only one who suffers and they understand what you are going through.”*

- *Gaining new knowledge*. N=42
  - Some spoke about general information N=10 others specifically about aspects of medication (N=11), about headache types and stages (N=10) and about new management strategies (N= 8) about triggers (N=3)
- *Component preferences* of the intervention. N=34
  - The most valued was relaxation (N=11), Mindfulness (N=7), Listening and communicating sessions (N=6) and Unhelpful thoughts (N=3), Facilitators (N=7)

**Question 10 What parts of the two day course, if any, did you least enjoy or find less valuable?**

55 participants responded. We have only presented the three main themes across the data concerning what was least enjoyable or valuable :

- *Timing and pitch of delivery (N=14 ).*
  - This included when people felt the days were too long, or too much or too little time was spent on different subjects and more activity rather than sitting for too long in front of a screen. Most were in the context of suggestions to improve it however some wanted more time and some wanted less.
- *Venue Environment:* ( N=12)
  - Many different things fell into this category including unhelpful lighting, heating, parking, poor ventilation feeling cramped noise levels or looking at a screen.
- *Component preferences* of the intervention
  - Mindfulness not clear or difficult (N=9), Relaxation unspecified (N=5)

The **Any other comments?** open question was responded to by 75 people. These have been classified into positive comments, negative comments and those which were not applicable to either of these responses.

Out of 75 participants’ comments 42 were positive which included praise or thanks for the course or the study and 13 negative, mainly to do with relevance and delivery and 20 not applicable.

We only had a response rate of 114/ 336 who attended the group intervention. It is unclear whether the trial participant’s satisfaction may have affected whether they returned their forms in the first place. It is also unclear whether those who had a positive or negative experience of the groups were more or less likely to be responders. The data is only from 114 participants so the results should be read in light of this. However we will be using this data alongside other sources of data such as attendance and attrition rates and staff delivery staff interviews which will provide triangulation of the experiences of the group overall.

On the whole the feedback looks as though the intervention was well received although some participants found specific components more or less personally valuable and some would advocate some changes to the content or delivery.
